# Ischemic Priapism Progressing to Penile Gangrene in a Patient with COVID-19 Infection: A Case Report with Literature Review

**DOI:** 10.1155/2022/8408216

**Published:** 2022-02-08

**Authors:** Saad M. Alsaedi, Rakan M. Alsarwani, Ahmed I. Ali, Saleem A. Aladhrai

**Affiliations:** ^1^College of Medicine, Umm Al-Qura University, Makkah, Saudi Arabia; ^2^Department of Urology, Security Forces Hospital Program, Makkah, Saudi Arabia; ^3^Department of Emergency Medicine, Security Forces Hospital Program, Makkah, Saudi Arabia

## Abstract

Priapism is considered a rare disorder and even more rare when it occurs as a complication of COVID-19. To the best of our knowledge, only eight studies have reported priapism as a complication of COVID-19. Here, we report the case of a 66-year-old male with COVID-19 who presented with neglected priapism for three days. On local examination, penile erection was apparent in association with blackened areas on the glans penis extending to the midpenile shaft denoting penile gangrene. A clear line of demarcation was noticed at the midpenile shaft. Penile duplex was performed, showing no blood flow in both cavernosal arteries. Penile aspiration was performed, and the cavernosal blood sample showed evidence of ischemic priapism. Given the presence of penile gangrene extending to the midshaft of the penis and the poor general condition of the patient, the decision was made to perform partial penectomy and suprapubic tube placement. We recommend the establishment of a guideline for the diagnosis and prevention of thrombotic diseases in patients with COVID-19 infection as there is increasing evidence of COVID-19-related thrombotic manifestations.

## 1. Introduction 

A novel coronavirus (CoV) emerged in Wuhan, China, at the beginning of December 2019, named “COVID-19” by the World Health Organization (WHO) [1]. It causes a range of respiratory and gastrointestinal symptoms, including fatigue, cough, and fever that may progress to severe respiratory failure [2]. Other life-threatening complications include venous and arterial thromboembolism [3]. Pulmonary embolism (PE) is recognized as the most common thrombotic manifestation, while arterial events have been reported less frequently [4]. Priapism is considered a rare disorder [5] and even more rare when it occurs as a complication of COVID-19. To the best of our knowledge, only eight studies have reported priapism as a complication of COVID-19 [6–13].

Here, we report a case of a 66-year-old male with COVID-19 who presented with neglected priapism for three days and penile gangrene.

## 2. Case Presentation

A 66-year-old male patient presented to the emergency room (ER) with shortness of breath, cough, and generalized fatigue for two days; he tested positive for COVID-19 infection. However, he had a stable O_2_ saturation and was discharged accordingly. Five days following discharge, the patient represented to the ER with shortness of breath and neglected priapism of three days duration.

On his second ER visit, his O_2_ sat was 78% on room air, 92-93% on 15 L/min nonrebreather mask, blood pressure was 126/81 mmHg, respiratory rate was 21/min, pulse rate was 110 beats per minute, and body temperature was 36.9°C. His Glasgow Coma Scale (GCS) score was 15 out of 15.

On examination, the patient was conscious and distressed. A priapism was observed together with blackened areas on the glans penis extending to the midpenile shaft denoting penile gangrene. A clear line of demarcation was noticed at the midpenile shaft.

The patient's medical history included type 2 diabetes mellitus (DM), hypertension (HTN), ischemic cardiomyopathy, and chronic kidney disease (CKD): stage 3b with baseline creatinine 1.5-1.6 mg/dL, atrial fibrillation, an old cerebrovascular accident (CVA) with no residual weakness apart from baseline expressive aphasia, and depression.

The patient's medications included amlodipine, insulin NovoMix, and hydralazine. He had also been taking warfarin 7.5 mg once daily and risperidone 0.25 mg and mirtazapine 30 mg once daily as needed for the past five years.

Laboratory findings at admission showed leukocytes of 10.70 × 10 e3/uL (4.00–11.00), hemoglobin 11.1 g/dL (13.5–17.2), platelet count 220 × 10 e9/L (150–450), prothrombin time (PT) 90 sec (11.0–15.0), international normalized ratio (INR) 7.19 (0.89–1.10), C-reactive protein (CRP) 219.50 mg/L (0.00–5.00), creatinine 244.20 umol/L (60.00–115.00), urea in serum 23.50 mmol/L (3.00–9.20), D-dimer 20.0 mg/L (0.0–0.5), and a positive COVID-19 polymerase chain reaction (PCR) test.

Penile duplex was performed and showed no blood flow in both cavernosal arteries. Magnetic resonance imaging (MRI) for the penis was requested to evaluate for necrosis but was unavailable in the hospital, and the patient could not be transferred due to his desaturation status.

### 2.1. Therapeutic Intervention

On admission, warfarin was withheld, and vitamin K 10 mg IV was administered together with four units of fresh frozen plasma (FFP) to correct his elevated INR. He also received dexamethasone 6 mg IV and ceftriaxone 2 gm IV infusions. The patient was then admitted under the intensive care unit/internal medicine (ICU/IM) for further management of his COVID-19 infection and warfarin toxicity.

A suprapubic tube was placed to relieve urinary retention by the urology team, who further recommended penile aspiration/surgical intervention after INR correction. Later, in the same day, the patient developed hypoxia and was connected to high flow nasal canula (HFNC): FLOW 60, FIO_2_ 100%, and stabilized. His INR had reduced from 7.14 to 1.70, and anesthesia consultation was done for surgical approval.

The patient was taken to the operating theatre (OR), and as he was very agitated and a trial of a local penile block was not feasible, general anesthesia was introduced. Penile aspiration was performed, and the cavernosal blood sample showed evidence of ischemic priapism. However, penile aspiration was not successful to alleviate his erection. Given the intraoperative findings and the patients' poor general condition, it was decided to perform a partial penectomy.

Postoperatively, he was kept intubated and mechanically ventilated for three days, at which point he was successfully extubated. Eventually, he was discharged home with O_2_ maintained on room air.

## 3. Discussion

Priapism is a penile erection that lasts four hours or longer and is unrelated to sexual stimulation [14]. Priapism is grouped into three types: ischemic type (which requires immediate clinical intervention), nonischemic type, and stuttering (recurrent) priapism. Ischemic priapism (also known as venoocclusive priapism) is characterized by a painful penile erection which persists for a prolonged time leading to little or no blood flow to the corporal bodies [15]. Priapism can result from conditions associated with increased blood viscosity such as sickle cell disease and hematological malignancies [16], and hyperviscosity in patients with COVID-19 has been previously reported in the literature [17, 18], possibly accounting for the development of priapism in COVID-19 patients.

Moreover, COVID-19 is known to cause hypercoagulability although the underlying mechanisms for this are not well understood. Hypotheses include cytokine storm, complement activation, shutdown of fibrinolysis, and COVID-19 itself activating the coagulation cascade. Excessive release of cytokines causes thrombosis through a variety of processes, including the activation of monocytes, neutrophils, and the endothelium. All of these mechanisms can contribute to the prothrombic state [4, 19]. It has been documented that COVID-19 patients can develop thrombotic events even when they are anticoagulated. Therefore, screening for thromboembolic events is essential in such patients [20]. In our literature review, we found eight cases of ischemic priapism, including one case of stuttering ischemia associated with COVID-19 infection (Table 1). Reported cases included patients ranging in age from 34 to 69 years, of whom one was also undergoing warfarin therapy, as in the current case. By comparison to our case, however, priapism did not progress to gangrene in any of the previously reported cases nor did any patient require surgery. No known risk factor for priapism was reported in four cases, and detumescence was achieved with an intracavernosal injection in five of the eight cases.

In our presented case, there were many risk factors for developing priapism other than COVID-19 infection, including unopposed warfarin action and antidepressant medication. However, it is only COVID-19 that had a recent onset in our patient compared to his long-term use of antidepressants and warfarin. Regardless of the cause, however, priapism rarely progresses to penile gangrene [21]. Other studies have also suggested that priapism was most likely induced by COVID-19 infection rather than other factors [6–8]. All of these evidences strongly suggest the need for the diagnosis and prevention of thrombotic diseases in at-risk patients with COVID-19 infection.

## Figures and Tables

**Table 1:** Documented cases of priapism related to COVID-19 infection in the literature.

Study	Age (y)	Type of priapism	Duration of priapism	ICU admission	Drug history	Medical history/comorbidities	Treatment of priapism	Follow-up/outcome	Purported priapism risk factors
Lam et al., 2020 [6]	67	Ischemic	Unknown duration	N/A	Warfarin	Dilated cardiomyopathy of unknown etiology, left bundle branch block, cutaneous scleroderma, paroxysmal atrial fibrillation, DM type 2, and iron deficiency anemia	Conservative	Died due to clinical deterioration	Minor trauma
									Warfarin
Silverman et al., 2021 [7]	69	Ischemic	Unknown duration (>3 hours)	Yes	N/A	Obesity	Intracavernosal phenylephrine injection	Achieved detumescence, but eventually died due to clinical deterioration	Propofol
Lamamri et al., 2021 [8]	62	Ischemic	Unknown duration (>4 hours)	Yes	None	Left inguinal surgery and appendectomy	Intracavernosal ethylephrine injection	Achieved detumescence and was discharged to ward	None
Addar et al., 2021 [9]	62	Ischemic	10 days	Yes	N/A	HTN and dyslipidemia	Intracavernosal phenylephrine injection	Achieved detumescence and was discharged home	None
Carreño et al., 2021 [10]	39	Ischemic	3 days	Yes	None	Overweight	Intracavernosal adrenaline injection	Failed to achieve detumescence and eventually died due to clinical deterioration	Propofol
Grimberg et al., 2021 [11]	45	Stuttering ischemic priapism	Unknown duration (>4 hours)	N/A	N/A	HTN and benign prostatic hyperplasia	Intracavernosal phenylephrine injection	Achieved detumescence, but had recurrence 8 h later and was managed with another intracavernosal phenylephrine injection. He achieved detumescence eventually with no recurrence afterward. He was discharged home	None
Larrarte-arenas et al., 2021 [12]	65	Ischemic	30 hours	No	Nifedipine, prazosin, calcitriol, subcutaneous erythropoietin, and unfractionated heparin	Chronic kidney disease on hemodialysis, HTN, secondary hyperparathyroidism, and anemia	Intracavernosal epinephrine injection	Achieved detumescence	Renal replacement therapy
									Prazosin
Giuliano et al., 2021 [13]	34	Ischemic	36 hours	No	None	None	Initially, intracavernosal phenylephrine injection (failed to achieve detumescence). Then, a bilateral T-shunt procedure was performed.	The bilateral T-shunt helped to achieve detumescence, and the patient was discharged home. However, it was complicated by complete erectile dysfunction after 3 months of follow-up	None
Our case	66	Ischemic priapism progressed to penile gangrene	3 days	Yes	Amlodipine, insulin NovoMix, hydralazine, warfarin, risperidone, and mirtazapine	DM type 2, HTN, ischemic cardiomyopathy, chronic kidney disease, atrial fibrillation, cerebrovascular accident, and depression	Partial penectomy	The patient improved and eventually was discharged home	Warfarin
									Antidepressant/antipsychotic

## Data Availability

The data used to support the findings of this study are available from the corresponding author upon request.
